# 20% Autologous serum vs. 0.05% cyclosporine and preservative-free artificial tears in the treatment of Sjögren related dry eye

**DOI:** 10.5935/0004-2749.2022-0192

**Published:** 2022-10-19

**Authors:** Mustafa Berhuni, Şeref İstek, Nesime Setge Tıskaoğlu

**Affiliations:** 1 Ersin Arslan Research and Education Hospital, Gaziantep, Turkey; 2 Antalya Kepez State Hospital, Antalya, Turkey

**Keywords:** Sjögren’s syndrome/complications, Dry eye syndrome/etiology, Dry eye syndrome/drug therapy, Cyclosporine/therapeutic use, Lubricant eye drops, Síndrome de Sjögren/complicações, Síndrome do olho seco/etiologia, Síndrome do olho seco/tratamento farmacológico, Ciclosporina/uso terapêutico, Lubrificantes oftalmológicos

## Abstract

**Purpose:**

To compare the 3-month results of treatment with 20% autologous serum or combination treatment with preservative-free artificial tears and 0.05% cyclosporine in patients with dry eye disease due to primary Sjögren’s syndrome.

**Methods:**

A total of 130 eyes of 65 patients with newly diagnosed dry eye disease due to primary Sjögren’s syndrome were included in the study. The patients were divided into two treatment groups: 66 eyes of 33 patients were assigned to the autologous serum treatment group, and 64 eyes of 32 patients were assigned to the combination treatment group. Schirmer test, tear break-up time and Ocular Surface Disease Index (OSDI) scores were recorded at pretreatment and at 3 months of treatment.

**Results:**

At 3 months of treatment, the mean Schirmer value and the mean tear break-up time were significantly higher in the combination treatment group (p<0.0001 and p=0.034, respectively). The OSDI score at 3 months was significantly lower in the autologous serum Group (p=0.004). When the two groups were evaluated separately, the improvements in Schirmer, tear break-up time test, and OSDI scores from before to after treatment were statistically significant: p<0.0001, p<0.001, and p<0.0001, respectively, for the authologus serum Group, and p<0.0001, p<0.001, and p<0.0001, respectively, for the combination treatment group.

**Conclusions:**

In short-term treatment of dry eye disease due to primary Sjögren’s syndrome, treatment with autologous serum was significantly superior to -combination treatment with preservative-free artificial tears and 0.05% cyclosporine in terms of improvement in OSDI scores. Improvements in Schirmer test and tear break-up time scores were significantly superior in the group treated with preservative-free artificial tears and 0.05% cyclosporine.

## Abstract (pt)

**Objetivo:**

Comparar os resultados de 3 meses de soro autólogo a 20% com um tratamento combinado, ou seja, lubrificantes oculares sem conservantes e ciclosporina a 0,05% em pacientes com síndrome do olho seco devida à síndrome de Sjögren primária.

**Métodos:**

Foram incluídos no estudo 130 olhos de 65 pacientes recentemente diagnosticados com síndrome do olho seco devida à síndrome de Sjögren primária. Os pacientes foram divididos em dois grupos de tratamento, 66 olhos de 33 pacientes foram incluídos no grupo de tratamento com soro autólogo e 64 olhos de 32 pacientes foram incluídos no grupo de tratamento combinado com lubrificantes oculares sem conservantes e ciclosporina. Os resultados do teste de Schirmer e do tempo de ruptura do filme lacrimal e os índices de doença da superfície ocular (OSDI) foram registrados antes e depois de três meses de tratamento.

**Resultados:**

Três meses após o tratamento, o valor médio do teste de Schirmer foi mais alto com significância estatística no grupo do tratamento combinado com lubrificantes oculares sem conservantes e ciclosporina (p<0,0001) e o tempo de ruptura do filme lacrimal também foi significativamente maior nesse grupo (p=0,034). Também aos três meses, a doença da superfície ocular foi menor com significância estatística no grupo de tratamento com soro autólogo (p=0,004). Quando os dois grupos foram avaliados separadamente, a melhora no teste de Schirmer, o tempo de ruptura e a doença da superfície ocular antes e depois do tratamento tiveram diferenças estatisticamente significativas tanto no grupo de soro autólogo (p<0,0001, p<0,001 e p<0,0001, respectivamente) quanto no grupo de tratamento combinado (p<0,0001, p<0,001 e p<0,0001, respectivamente).

**Conclusões:**

No tratamento de curto prazo da síndrome do olho seco devida à síndrome de Sjögren primária, o tratamento com soro autólogo foi significativamente superior ao tratamento com lubrificantes oculares sem conservantes combinados com ciclosporina, em termos de melhora no doença da superfície ocular. As melhoras no teste de Schirmer e no tempo de ruptura do filme lacrimal foram significativamente maiores no grupo de tratamento combinado com lubrificantes oculares sem conservantes e ciclosporina.

## INTRODUCTION

Dry eye disease (DED) is a multifactorial disease that presents with ocular surface signs and subjective symptoms such as visual impairment, burning, stinging, foreign body sensation, and itching. In the most recent International Dry Eye Workshop report, DED was defined as a multifactorial tear and ocular surface disease with symptoms of discomfort and tear film instability^(1)^. Tear film instability, increased tear osmolarity, and associated ocular surface inflammation are the most common findings^(2)^.

DED has an increased incidence with age and is predominantly seen in women^(3,4)^. DED can be classified into three groups according to the underlying mechanism: decreased aqueous production due to disorder in the lacrimal glands, increased evaporation due to meibo mian gland dysfunction, and mixed type^(5,6)^. The most important systemic disease causing aqueousdeficient dry eye is Sjögren’s syndrome, a systemic autoimmune inflammatory disease characterized by dry eye and dry mouth^(5)^. Decreased tear secretion together with inflammation seen in Sjögren’s disease can lead to complications such as corneal scarring, sterile corneal ulceration, and even corneal perforation^(1)^. Effective treatment of these patients is needed to avoid the “vicious cycle” in which tear instability leads to tear hyperosmolarity, apoptosis and inflammation^(6)^.

Topical treatment regimens for Sjögren-related DED consist of lubricants, cyclosporine ophthalmic emulsion (COE), corticosteroids, nonsteroidal anti-inflammatory drugs, autologous serum (AS), and tacrolimus^(5)^. The side effects and safety profiles of the main agents used, COE, AS, and preservative-free artificial tears (PFAT), are superior^(7)^. AS was first used in the treatment of ocular alkaline burns by Ralph et al.^(8)^. The chemical composition of AS is similar to that of tears secreted from the lacrimal gland, including growth factors, proteins, and various nutrients^(9)^. Many studies have demonstrated the efficacy of AS eye drops in ocular surface disorders such as DED, persistent epithelial defects, neurotrophic keratopathy, superior limbic keratoconjunctivitis, and chemical burns^(10)^.

The discovery that inflammatory factors play an important role in DED has led to the increased therapeutic use of anti-inflammatory agents^(7)^. A phase 3 study conducted in 2002 demonstrated the efficacy and safety of COE in moderate and severe DED. COE has both immunomodulatory and anti-inflammatory effects as well as minimal side effects compared with corticosteroids^(11)^. It exerts its anti-inflammatory effects by inhibiting the activation of T cells in the conjunctiva and inhibiting the associated transcription of interleukin 2^(12)^. Studies have shown that 0.05% cyclosporine increases tear secretion in moderate and severe DED and improves Schirmer scores, tear break-up time (TBUT), ocular staining scores, conjunctival goblet cell density, and OSDI scores^(13)^.

In this study, we aimed to compare the 3-month results of 20% AS with combination treatment, i.e., PFAT and COE, in patients with DED due to primary Sjögren’s syndrome (PSS).

## METHODS

This study was a prospective, randomized, controlled, clinical trial. The study was performed at Antalya Kepez State Hospital between June 2017 and June 2019. A total of 130 eyes of 65 patients (96 eyes of 48 women and 34 eyes of 17 men) with newly diagnosed DED due to PSS were included in the study. The study was carried out in accordance with the principles of the Declaration of Helsinki and was approved by the Institutional Review Board of the Erciyes University Ethical Committee for Clinical Investigations (approval number: 2016/548). Informed consent was obtained from each patient included in the study.

The patients were divided into two treatment groups: 33 patients to the AS treatment group and 32 patients to the PFAT-COE combination treatment group. Sixty-six eyes of the 33 patients in the AS group received one drop of 20% AS 6 times a day; 64 eyes of the 32 patients in the combination group received one drop of COE (Restasis®; Allergan, Inc., Irvine, CA) twice a day and one drop of PFAT (Refresh® Single Dose Preservative Free, Allergan Inc.) 6 times a day. Detailed eye examinations, including best corrected visual acuity (BCVA), intraocular pressure (IOP) measurements, and anterior and posterior segment evaluation were performed. The Schirmer test and TBUT were performed in the morning by a single examiner (SI), who was blinded to the status of the patients.

TBUT was assessed using a fluorescein strip (Visimed, Izmir, Turkey), which was moistened and placed into the lateral one-third of the lower eyelid, and the interval between the last complete blink and the appearance of the first corneal black spot in the stained tear film was measured. Three separate measurements were taken, and the average of the measurements was recorded. The Schirmer test was performed without anesthetic by placing the strip into the lower conjunctival sac at the junction of the lateral and middle thirds, and the length of wetting was recorded after 5 minutes. Dry eye symptoms were assessed by the Ocular Surface Disease Index (OSDI), a 12-item questionnaire developed before the ophthalmologic examinations.

DED was defined as Schirmer I Test (without topical anesthesia) <10 mm, TBUT <10 s, and OSDI score ≥23. The Schirmer, TBUT, and OSDI tests were performed before treatment and after 3 months of treatment in both groups.

For preparation of AS, 15 mL of venous blood was drawn using a vacutainer and centrifuged at 4000 rpm for 10 minutes. The centrifuged serum was withdrawn from the blood tubes under sterile conditions and diluted with 0.9% NaCl solution to obtain a 20% concentration. The AS was placed in 15-mL bottles and wrapped with aluminum foil to protect the vitamin A from ultraviolet light. All patients were asked to keep the prepared AS eye drops at 4°C.

### Exclusion criteria

Patients with active ocular infection, any inflammatory condition not associated with DED, ocular allergy, eyelid or eyelash abnormality, contact lens use, history of refractive surgery, glaucoma, or use of topical eye drops were excluded from the study. Patients with graft- versushost disease, severe anemia (hemoglobin <11 g/dL), significant medically uncontrolled cerebrovascular or cardiovascular disease or who were pregnant were also excluded.

Patients with PSS diagnosed with DED diagnostic tests (Schirmer <10 mm/5 min without anesthesia, TBUT <10 s, OSDI >23) and patients with BCVA or uncorrected visual distance acuity (UCVDA) of 20/20 were included in the study.

Statistical analyses were performed using SPSS version 15.0 for Windows Evaluation Version release (SPSS, Chicago, IL). Descriptive statistics were presented as mean ± SD for the variables (OSDI score, Schirmer’s Test I, and TBUT). All the variables were normally distributed (Kolmogorov-Smirnov and Shapiro-Wilk tests). Student’s paired samples *t*-test and independent samples *t*-test were used to compare data. All analyses were performed with a power of 80% and 95% CI. The level of statistical significance was set at p<0.05.

## RESULTS

The mean age of the patients was 54.04 ± 7.94 years. The demographic and initial characteristics of the patients are shown in table 1. The mean Schirmer, TBUT, and OSDI scores of all patients before treatment were 7.61 ± 3.70, 4.91 ± 2.24, and 75.03 ± 12.37, respectively. The mean Schirmer, TBUT, and OSDI scores of patients in the AS group were 6.77 ± 3.07, 4.44 ± 1.5, and 74.69 ± 11.87, respectively, and 6.33 ± 3.32, 4.11 ± 1.32, and 79.70 ± 12.37 for patients in the COE-PFAT group. There were no statistically significant differences between the groups in any of the tests (p0.345, p=0.197, p=0.620, respectively).

**Table 1:** Demographic characteristics and baseline measurements of patients

Total no. of patients (eyes)	65 (130)
Sex–no. (%)	
Female	96 (73.8)
Male	34 (26.2)
Mean±SD age (yr)	54.04±7.94
Mean±SD ST-1 (mm)	7.61±3.70
Mean±SD TBUT (s)	4.91±2.24
Mean±SD OSDI score	75.03±12.37

ST-1= schrimer test 1; TBUT= tear break-up time; OSDI= Ocular Surface Disease Index.

At 3 months of treatment, the mean Schirmer value was 8.83 mm in the AS group and 18 mm in the COE-PFAT group and was significantly higher in the COE-PFAT group (p<0.0001). At 3 months of treatment, the TBUT value was 5.94 s in the AS group and 6.88 s in the COE-PFAT group and was significantly higher in the COE-PFAT group (p=0.034). The OSDI score at 3 months of treatment was 27.72 in the AS group and 37.30 in the COE-PFAT group and was significantly lower in the AS group (p=0.004). When the two groups were evaluated separately, the improvements in Schirmer, TBUT, and OSDI scores from before to after treatment were statistically significant in the AS Group (p<0.0001, p<0.001, p<0.0001, respectively) and the PFAT Group (p<0.0001, p<0.001, p<0.0001 respectively) (Table 2).

**Table 2:** Comparison of mean OSDI, TBUT and ST-1 measurement pre-treatment and postreatment between AS and COE-PFAT groups

	Pretreatment	Post-treatment	p value
Mean ± SD OSDI score			
AS group	74.69 ± 11.87	27.72 ± 6.62	p<0.0001*
COE-PFAT group	79.70 ± 12.37	37.30 ± 7.92	p<0.0001*
p value	p=0.197**	p:0.004**	
Mean ± SD TBUT (s)			
AS group	4.44 ± 1.5	5.94 ± 1.69	p<0.001*
COE-PFAT group	4.11 ± 1.32	6.88 ± 2.11	p<0.001*
p value	p=0.345**	p=0.034**	
Mean ± SD ST-1 (mm)			
AS group	6.77 ± 3.07	8.83 ± 3.32	p<0.0001*
COE-PFAT group	6.33 ± 3.32	18 ± 7.94	p<0.0001*
p value	p=0.620**	p<0.0001**	

*= Student’s paired samples *t*-test, **= independent samples Student’s *t*-test. AS= autologous serum; COE= cyclosporine ophthalmic emulsion; PFAT= preservative-free artificial tears; TBUT= tear break-up time; OSDI= Ocular Surface Disease Index; ST-1= schrimer test-1.

## DISCUSSION

Since AS was first described by Ralph et al.^(7)^, it has been used in many studies of severe and treatment-resistant DED^(14,15)^. In patients with severe DED refractory to conventional therapy, AS eye drops are used to reduce inflammation and improve symptoms and signs^(16)^. AS has properties similar to those of normal tears, as it contains nutrients, proteins, and growth factors that reduce inflammation and protect the eye^(17)^. Studies have shown the effectiveness of AS in the treatment of Sjögren’s syndrome, neurotrophic keratopathy, and persistent corneal epithelial defect^(18)^.

COE is an anti-inflammatory and immunomodulatory agent and is used in the long-term treatment of patients with chronic moderate and severe DED^(19)^. Many studies have shown that it effectively improves the symptoms and signs of DED^(19,20)^. Previous studies have compared the effectiveness of treatment with different concentrations of AS with treatment with artificial tears in DED^(21,22,23)^. To the best of our knowledge, this is the first study comparing the 3-month treatment results of 20% AS and COE-PFAT in DED due to PSS.

Kojima et al., in their study comparing the 2-week results of AS and AT treatments in DED, reported that there was no significant difference between the two groups in Schirmer scores; however, there was a statistically significant increase in TBUT in the AS group(^18^). Noble et al. compared the 3-month results of 50% AS and AT treatments in patients with diseases that cause ocular surface disorders, i.e., Sjögren’s syndrome, keratoconjunctivitis sicca, graft-versus-host disease, and cicatricial pemphigoid, and found no significant differences between the groups in Schirmer and TBUT values(^21^).

Urzua et al. found no significant difference in TBUT in their study comparing the 2-week results of 20% AS and AT treatment in 12 patients with severe DED; they argued that this result might have been due to the short follow-up period(^24^). A study by Celebi et al. comparing the 1-month results of 20% AS and AT treatments in 40 eyes of 20 patients with severe DED found no significant difference between the two groups in Schirmer scores, but a significant increase in TBUT in the AS group(^25^).

In a study by Yılmaz et al. of the 1-month results of 40% AS and AT in patients with DED due to systemic isotretinoin use, TBUT was significantly higher in the AS group than in the AT group. The authors noted that there was no significant difference in Schirmer scores(^23^). In our study, improvements in Schirmer and TBUT scores after 3 months of treatment were significantly higher in the COE-PFAT group than in the AS group.

COE is known to increase basal tear secretion, which would explain the higher Schirmer scores in the COE-PFAT group; additional lubrication with artificial tears would lead to the regulation of lacrimal gland secretion(^6^). TBUT measures tear film instability, which is most commonly linked to evaporative DED; COE has been shown to increase goblet cell density, thus preventing evaporative disease(^10^). The anti-inflammatory and anti-apoptotic properties of COE are well documented. Whatever the underlying pathogenesis, increase in tear osmolarity leads to inflammation that can trigger a vicious cycle(^10^). Our study suggests that the COE-PFAT combination may have superior anti-inflammatory properties to AS alone.

The OSDI questionnaire has been recommended by the International Dry Eye Workshop to measure symptomatic improvement in DED(^1^). Çelebi et al. found that after 1 month of treatment, the OSDI scores decreased from 55.45 to 25.85 (55.18% reduction) in the AS group and from 55.25 to 44.48 (19.5% reduction) in the PFAT group, and symptomatic improvement in the AS group was highly significant (p<0.001)^(25)^. In a study by Urzua et al., the OSDI score after 2 weeks of treatment was significantly lower in the AS group than in the AT group (p=0.002)^(24)^. Similarly, Yilmaz et al. found that the OSDI score was significantly lower in the AS group than in the AT group after 1 month of treatment (p<0.001)^(23)^. Similar to these studies, in our study, the OSDI score decreased from 74.69 to 27.72 (62.88% reduction) in the AS group and from 79.70 to 37.30 (53.19% reduction) in the COE-PFAT Group after 3 months of treatment, and symptomatic relief was significantly greater in the AS group than in the COE-PFAT group (p=0.004). Studies have shown that there is a discrepancy between the efficacy of treatment of dry eye signs and symptoms; whereas the COE-PFAT combination was more effective in treating the signs of DED in our patients, AS was superior in symptomatic relief. Studies of DED have shown a decrease in growth factors in tear secretion^(25)^, as AS contains growth factors that increase tear stability; this could be a reason for the superior relief seen with AS.

The limitations of our study were the lack of classification of the severity of DED before and after treatment, the small sample size, and the short duration of follow-up. No side effects of the treatment were observed in any of the patients.

In the short-term treatment of DED due to PSS, AS was significantly superior to PFAT-COE treatment in improvement of OSDI scores, which are indicative of symptomatic improvement. COE-PFAT treatment was significantly superior to AS treatment in improvements in Schirmer and TBUT scores, i.e., increased tear secretion and improvement of tear film stability. Studies with larger populations and longer follow-up periods are needed to compare the efficacy of AS and COE-PFAT in the treatment of dry eye associated with PSS.
